# Supplementary material for: Comprehensive microRNA analysis across genome-edited colorectal cancer organoid models reveals miR-24 as a candidate regulator of cell survival
Source: BMC Genomics. 2022 Dec 1;23:792. doi: 10.1186/s12864-022-09018-1 (PMC9716731; doi:10.1186/s12864-022-09018-1)
Supplement: Supplementary file 1 — Additional file 1: TableS1. Mapping statistics of small RNA-seq data produced from genetically modified mouse enteroids and WT controls. TableS2. Mapping statistics of RNA-seq data produced from genetically modified mouse enteroids and WT controls. TableS3. Differentially expressed miRNAs when comparing TCGA tumors with specific genotypes to non-tumor (NT) controls(>500 RPMMM in either condition, p-value<0.05, hochberg p-adj<0.2, and fold change >1.5x) and mutant mouse enteroids with WT control (baseMean>500DESeqnormalized counts, p-value<0.05, DESeq2 p-adj<0.2, and fold change>1.5x). MiRNAs in red are upregulated. MiRNAs in blue are downregulated. TableS4. Mapping statistics of RNA-seq data produced from HCT116 cells transfected with scramble control or miR-24 inhibitor. Table S5. 70 predicted miR-24-3p target genes (expression>500 normalized counts in either condition, p-adj<0.05, Fold change >0, predicted target by TargetScan) in response to miR-24-3p inhibition, relative to scamble, in HCT116 cells. TableS6. Mapping statistics of ChRO-seq data produced from HCT116 cells transfected with scramble control or miR-24inhibitor. Supplemental Figure 1. Heatmaps show the magnitude of change in miRNA expression relative to WT by subtracting rlog normalized miRNA expression for each enteroid sample by the rlog average WT expression. Groups not shown in the main text shown here. Color intensity rlog normalized miRNA expression in each genetically modified enteroid sample subtracted from average WT. Color scale minimum saturates at -3 and maximum saturates at 3. Supplemental Figure 2. (A)Brightfield images of mouse enteroids treated with 0, 0.5, or 1 ng/mL recombinant human TGF-B1. (B) Col1a1 and(C) Fn1 CTs from RT-qPCR. In cases for which gene expression was not detected at 40 cycles, CT was set to 40 for analysis. Significance determined by two-sided Wilcoxon test. *p<0.05, **p<0.01, ***p<0.001. Supplemental Figure 3. (A) PCA plot generated using gene expression profiles [file 12864_2022_9018_MOESM1_ESM.pptx]

## Slide 1
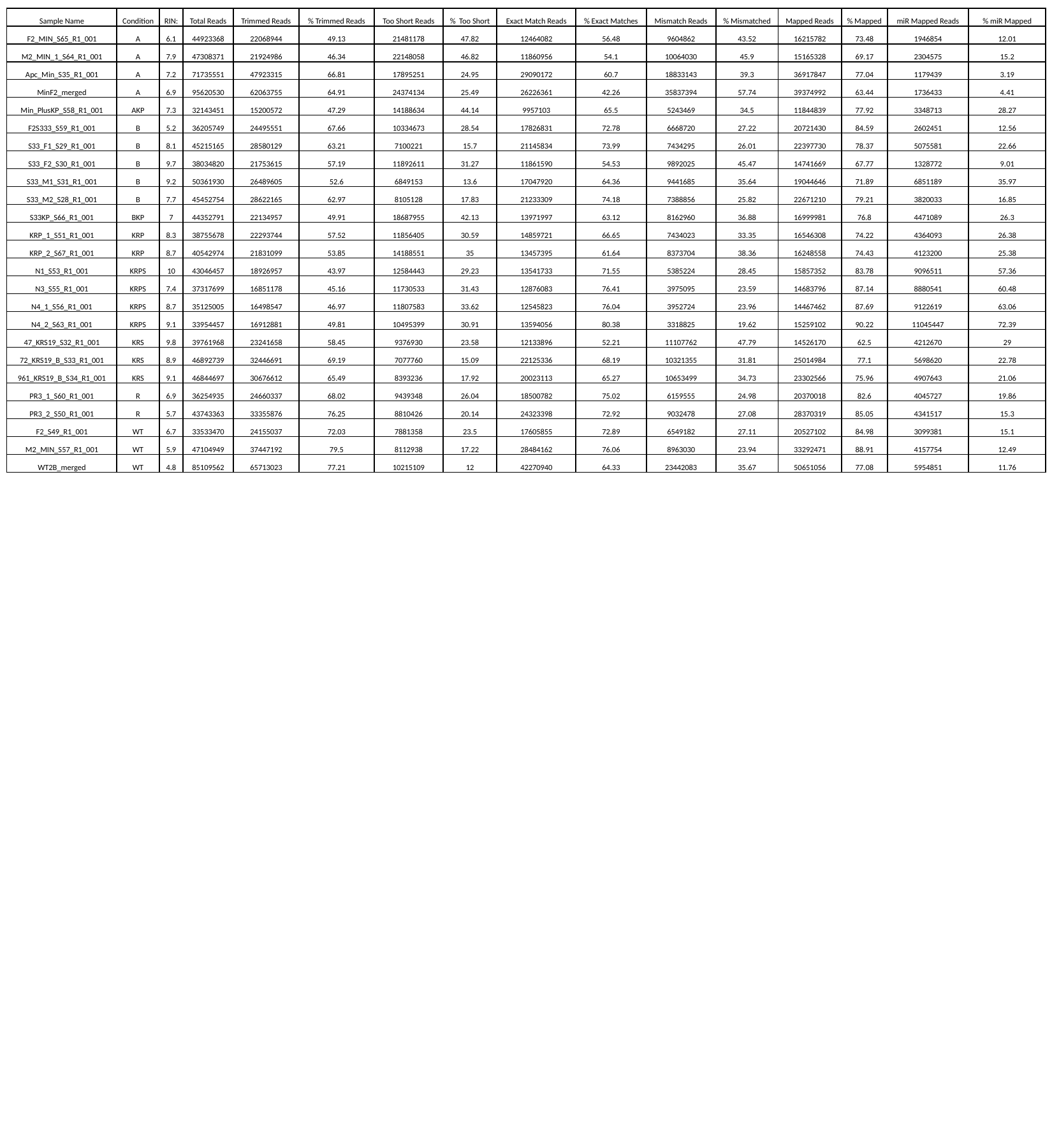

| Sample Name | Condition | RIN: | Total Reads | Trimmed Reads | % Trimmed Reads | Too Short Reads | % Too Short | Exact Match Reads | % Exact Matches | Mismatch Reads | % Mismatched | Mapped Reads | % Mapped | miR Mapped Reads | % miR Mapped |
| --- | --- | --- | --- | --- | --- | --- | --- | --- | --- | --- | --- | --- | --- | --- | --- |
| F2\_MIN\_S65\_R1\_001 | A | 6.1 | 44923368 | 22068944 | 49.13 | 21481178 | 47.82 | 12464082 | 56.48 | 9604862 | 43.52 | 16215782 | 73.48 | 1946854 | 12.01 |
| M2\_MIN\_1\_S64\_R1\_001 | A | 7.9 | 47308371 | 21924986 | 46.34 | 22148058 | 46.82 | 11860956 | 54.1 | 10064030 | 45.9 | 15165328 | 69.17 | 2304575 | 15.2 |
| Apc\_Min\_S35\_R1\_001 | A | 7.2 | 71735551 | 47923315 | 66.81 | 17895251 | 24.95 | 29090172 | 60.7 | 18833143 | 39.3 | 36917847 | 77.04 | 1179439 | 3.19 |
| MinF2\_merged | A | 6.9 | 95620530 | 62063755 | 64.91 | 24374134 | 25.49 | 26226361 | 42.26 | 35837394 | 57.74 | 39374992 | 63.44 | 1736433 | 4.41 |
| Min\_PlusKP\_S58\_R1\_001 | AKP | 7.3 | 32143451 | 15200572 | 47.29 | 14188634 | 44.14 | 9957103 | 65.5 | 5243469 | 34.5 | 11844839 | 77.92 | 3348713 | 28.27 |
| F2S333\_S59\_R1\_001 | B | 5.2 | 36205749 | 24495551 | 67.66 | 10334673 | 28.54 | 17826831 | 72.78 | 6668720 | 27.22 | 20721430 | 84.59 | 2602451 | 12.56 |
| S33\_F1\_S29\_R1\_001 | B | 8.1 | 45215165 | 28580129 | 63.21 | 7100221 | 15.7 | 21145834 | 73.99 | 7434295 | 26.01 | 22397730 | 78.37 | 5075581 | 22.66 |
| S33\_F2\_S30\_R1\_001 | B | 9.7 | 38034820 | 21753615 | 57.19 | 11892611 | 31.27 | 11861590 | 54.53 | 9892025 | 45.47 | 14741669 | 67.77 | 1328772 | 9.01 |
| S33\_M1\_S31\_R1\_001 | B | 9.2 | 50361930 | 26489605 | 52.6 | 6849153 | 13.6 | 17047920 | 64.36 | 9441685 | 35.64 | 19044646 | 71.89 | 6851189 | 35.97 |
| S33\_M2\_S28\_R1\_001 | B | 7.7 | 45452754 | 28622165 | 62.97 | 8105128 | 17.83 | 21233309 | 74.18 | 7388856 | 25.82 | 22671210 | 79.21 | 3820033 | 16.85 |
| S33KP\_S66\_R1\_001 | BKP | 7 | 44352791 | 22134957 | 49.91 | 18687955 | 42.13 | 13971997 | 63.12 | 8162960 | 36.88 | 16999981 | 76.8 | 4471089 | 26.3 |
| KRP\_1\_S51\_R1\_001 | KRP | 8.3 | 38755678 | 22293744 | 57.52 | 11856405 | 30.59 | 14859721 | 66.65 | 7434023 | 33.35 | 16546308 | 74.22 | 4364093 | 26.38 |
| KRP\_2\_S67\_R1\_001 | KRP | 8.7 | 40542974 | 21831099 | 53.85 | 14188551 | 35 | 13457395 | 61.64 | 8373704 | 38.36 | 16248558 | 74.43 | 4123200 | 25.38 |
| N1\_S53\_R1\_001 | KRPS | 10 | 43046457 | 18926957 | 43.97 | 12584443 | 29.23 | 13541733 | 71.55 | 5385224 | 28.45 | 15857352 | 83.78 | 9096511 | 57.36 |
| N3\_S55\_R1\_001 | KRPS | 7.4 | 37317699 | 16851178 | 45.16 | 11730533 | 31.43 | 12876083 | 76.41 | 3975095 | 23.59 | 14683796 | 87.14 | 8880541 | 60.48 |
| N4\_1\_S56\_R1\_001 | KRPS | 8.7 | 35125005 | 16498547 | 46.97 | 11807583 | 33.62 | 12545823 | 76.04 | 3952724 | 23.96 | 14467462 | 87.69 | 9122619 | 63.06 |
| N4\_2\_S63\_R1\_001 | KRPS | 9.1 | 33954457 | 16912881 | 49.81 | 10495399 | 30.91 | 13594056 | 80.38 | 3318825 | 19.62 | 15259102 | 90.22 | 11045447 | 72.39 |
| 47\_KRS19\_S32\_R1\_001 | KRS | 9.8 | 39761968 | 23241658 | 58.45 | 9376930 | 23.58 | 12133896 | 52.21 | 11107762 | 47.79 | 14526170 | 62.5 | 4212670 | 29 |
| 72\_KRS19\_B\_S33\_R1\_001 | KRS | 8.9 | 46892739 | 32446691 | 69.19 | 7077760 | 15.09 | 22125336 | 68.19 | 10321355 | 31.81 | 25014984 | 77.1 | 5698620 | 22.78 |
| 961\_KRS19\_B\_S34\_R1\_001 | KRS | 9.1 | 46844697 | 30676612 | 65.49 | 8393236 | 17.92 | 20023113 | 65.27 | 10653499 | 34.73 | 23302566 | 75.96 | 4907643 | 21.06 |
| PR3\_1\_S60\_R1\_001 | R | 6.9 | 36254935 | 24660337 | 68.02 | 9439348 | 26.04 | 18500782 | 75.02 | 6159555 | 24.98 | 20370018 | 82.6 | 4045727 | 19.86 |
| PR3\_2\_S50\_R1\_001 | R | 5.7 | 43743363 | 33355876 | 76.25 | 8810426 | 20.14 | 24323398 | 72.92 | 9032478 | 27.08 | 28370319 | 85.05 | 4341517 | 15.3 |
| F2\_S49\_R1\_001 | WT | 6.7 | 33533470 | 24155037 | 72.03 | 7881358 | 23.5 | 17605855 | 72.89 | 6549182 | 27.11 | 20527102 | 84.98 | 3099381 | 15.1 |
| M2\_MIN\_S57\_R1\_001 | WT | 5.9 | 47104949 | 37447192 | 79.5 | 8112938 | 17.22 | 28484162 | 76.06 | 8963030 | 23.94 | 33292471 | 88.91 | 4157754 | 12.49 |
| WT2B\_merged | WT | 4.8 | 85109562 | 65713023 | 77.21 | 10215109 | 12 | 42270940 | 64.33 | 23442083 | 35.67 | 50651056 | 77.08 | 5954851 | 11.76 |

## Slide 2
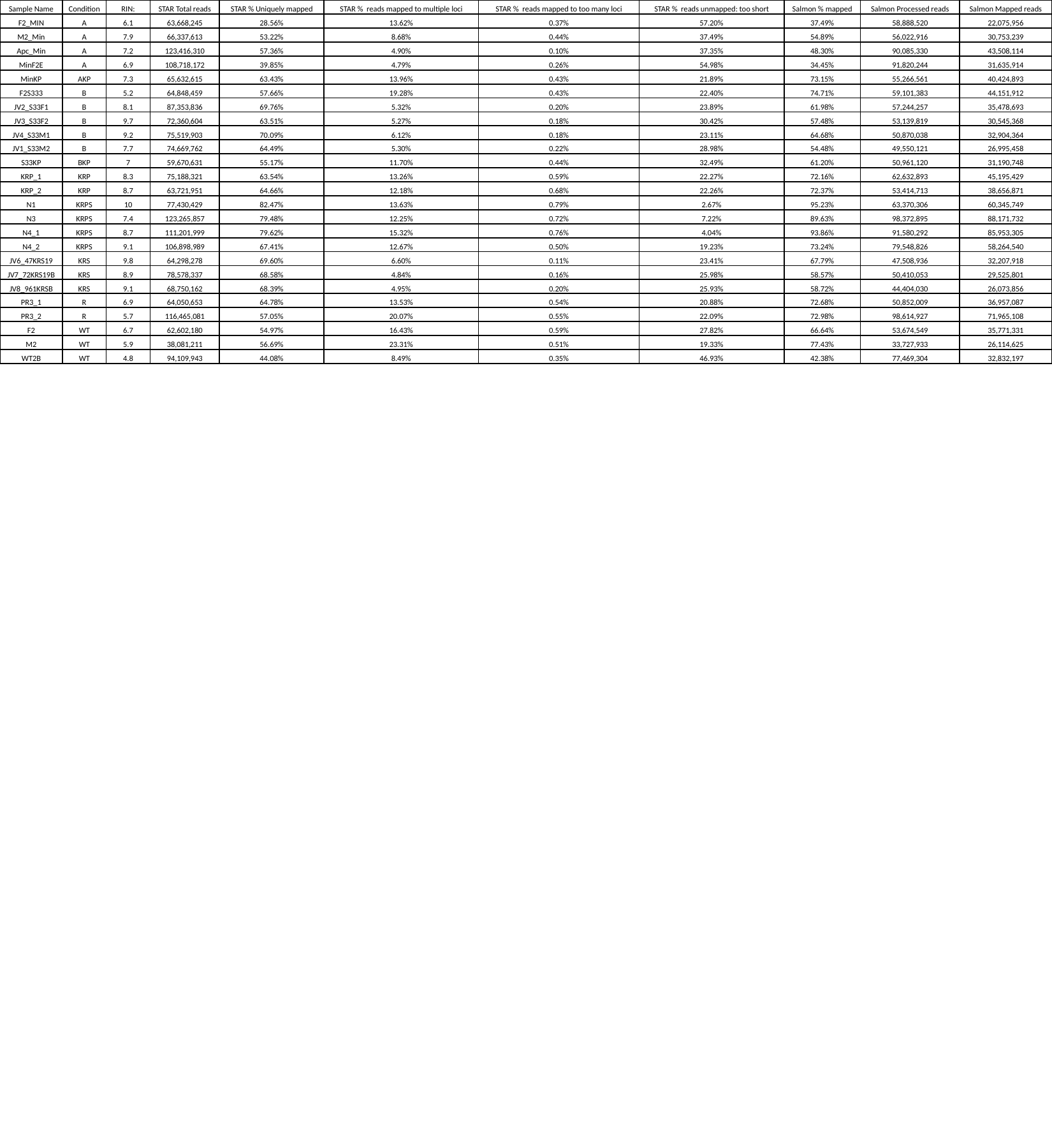

| Sample Name | Condition | RIN: | STAR Total reads | STAR % Uniquely mapped | STAR % reads mapped to multiple loci | STAR % reads mapped to too many loci | STAR % reads unmapped: too short | Salmon % mapped | Salmon Processed reads | Salmon Mapped reads |
| --- | --- | --- | --- | --- | --- | --- | --- | --- | --- | --- |
| F2\_MIN | A | 6.1 | 63,668,245 | 28.56% | 13.62% | 0.37% | 57.20% | 37.49% | 58,888,520 | 22,075,956 |
| M2\_Min | A | 7.9 | 66,337,613 | 53.22% | 8.68% | 0.44% | 37.49% | 54.89% | 56,022,916 | 30,753,239 |
| Apc\_Min | A | 7.2 | 123,416,310 | 57.36% | 4.90% | 0.10% | 37.35% | 48.30% | 90,085,330 | 43,508,114 |
| MinF2E | A | 6.9 | 108,718,172 | 39.85% | 4.79% | 0.26% | 54.98% | 34.45% | 91,820,244 | 31,635,914 |
| MinKP | AKP | 7.3 | 65,632,615 | 63.43% | 13.96% | 0.43% | 21.89% | 73.15% | 55,266,561 | 40,424,893 |
| F2S333 | B | 5.2 | 64,848,459 | 57.66% | 19.28% | 0.43% | 22.40% | 74.71% | 59,101,383 | 44,151,912 |
| JV2\_S33F1 | B | 8.1 | 87,353,836 | 69.76% | 5.32% | 0.20% | 23.89% | 61.98% | 57,244,257 | 35,478,693 |
| JV3\_S33F2 | B | 9.7 | 72,360,604 | 63.51% | 5.27% | 0.18% | 30.42% | 57.48% | 53,139,819 | 30,545,368 |
| JV4\_S33M1 | B | 9.2 | 75,519,903 | 70.09% | 6.12% | 0.18% | 23.11% | 64.68% | 50,870,038 | 32,904,364 |
| JV1\_S33M2 | B | 7.7 | 74,669,762 | 64.49% | 5.30% | 0.22% | 28.98% | 54.48% | 49,550,121 | 26,995,458 |
| S33KP | BKP | 7 | 59,670,631 | 55.17% | 11.70% | 0.44% | 32.49% | 61.20% | 50,961,120 | 31,190,748 |
| KRP\_1 | KRP | 8.3 | 75,188,321 | 63.54% | 13.26% | 0.59% | 22.27% | 72.16% | 62,632,893 | 45,195,429 |
| KRP\_2 | KRP | 8.7 | 63,721,951 | 64.66% | 12.18% | 0.68% | 22.26% | 72.37% | 53,414,713 | 38,656,871 |
| N1 | KRPS | 10 | 77,430,429 | 82.47% | 13.63% | 0.79% | 2.67% | 95.23% | 63,370,306 | 60,345,749 |
| N3 | KRPS | 7.4 | 123,265,857 | 79.48% | 12.25% | 0.72% | 7.22% | 89.63% | 98,372,895 | 88,171,732 |
| N4\_1 | KRPS | 8.7 | 111,201,999 | 79.62% | 15.32% | 0.76% | 4.04% | 93.86% | 91,580,292 | 85,953,305 |
| N4\_2 | KRPS | 9.1 | 106,898,989 | 67.41% | 12.67% | 0.50% | 19.23% | 73.24% | 79,548,826 | 58,264,540 |
| JV6\_47KRS19 | KRS | 9.8 | 64,298,278 | 69.60% | 6.60% | 0.11% | 23.41% | 67.79% | 47,508,936 | 32,207,918 |
| JV7\_72KRS19B | KRS | 8.9 | 78,578,337 | 68.58% | 4.84% | 0.16% | 25.98% | 58.57% | 50,410,053 | 29,525,801 |
| JV8\_961KRSB | KRS | 9.1 | 68,750,162 | 68.39% | 4.95% | 0.20% | 25.93% | 58.72% | 44,404,030 | 26,073,856 |
| PR3\_1 | R | 6.9 | 64,050,653 | 64.78% | 13.53% | 0.54% | 20.88% | 72.68% | 50,852,009 | 36,957,087 |
| PR3\_2 | R | 5.7 | 116,465,081 | 57.05% | 20.07% | 0.55% | 22.09% | 72.98% | 98,614,927 | 71,965,108 |
| F2 | WT | 6.7 | 62,602,180 | 54.97% | 16.43% | 0.59% | 27.82% | 66.64% | 53,674,549 | 35,771,331 |
| M2 | WT | 5.9 | 38,081,211 | 56.69% | 23.31% | 0.51% | 19.33% | 77.43% | 33,727,933 | 26,114,625 |
| WT2B | WT | 4.8 | 94,109,943 | 44.08% | 8.49% | 0.35% | 46.93% | 42.38% | 77,469,304 | 32,832,197 |

## Slide 3
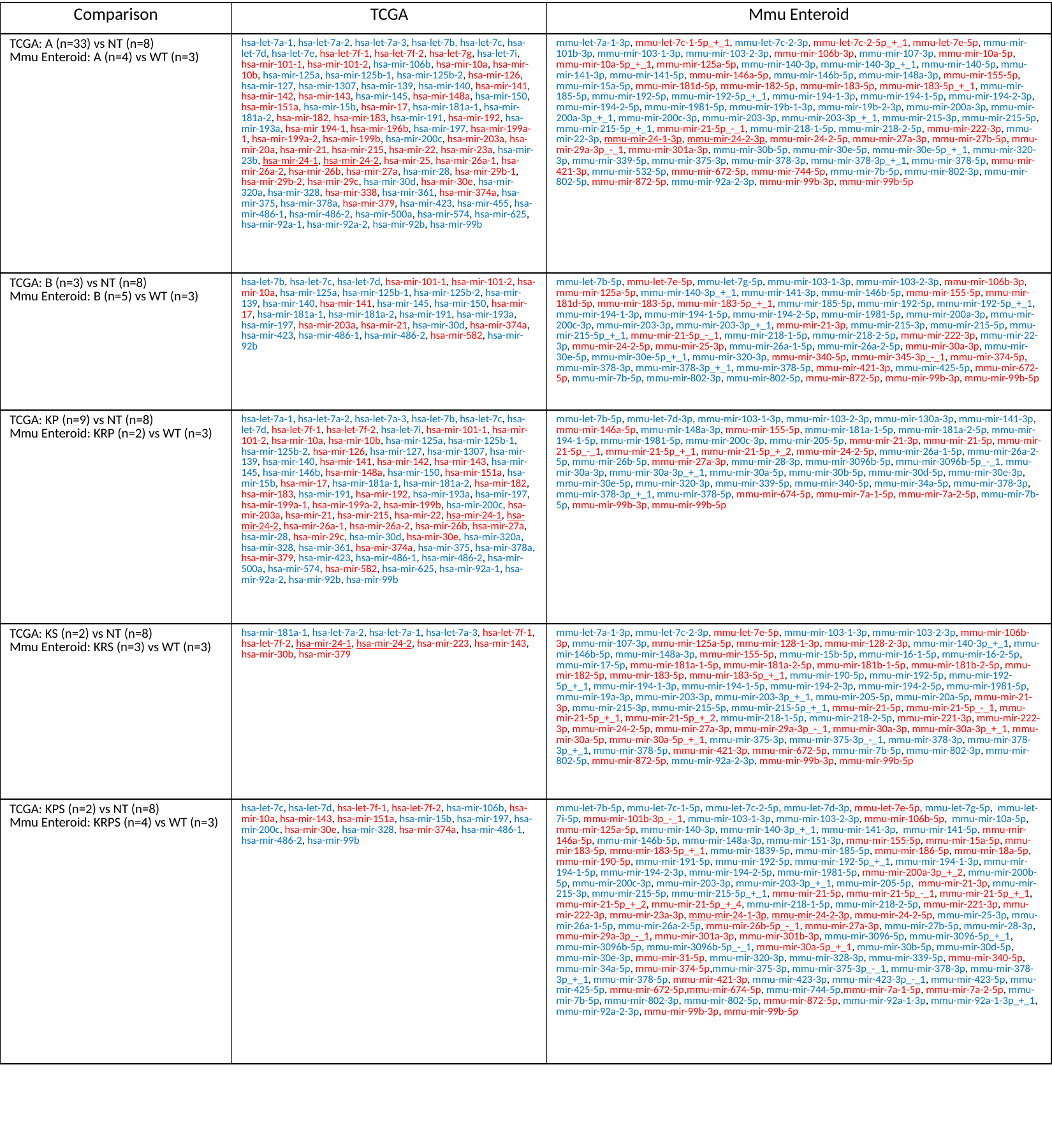

| Comparison | TCGA | Mmu Enteroid |
| --- | --- | --- |
| TCGA: A (n=33) vs NT (n=8) Mmu Enteroid: A (n=4) vs WT (n=3) | hsa-let-7a-1, hsa-let-7a-2, hsa-let-7a-3, hsa-let-7b, hsa-let-7c, hsa-let-7d, hsa-let-7e, hsa-let-7f-1, hsa-let-7f-2, hsa-let-7g, hsa-let-7i, hsa-mir-101-1, hsa-mir-101-2, hsa-mir-106b, hsa-mir-10a, hsa-mir-10b, hsa-mir-125a, hsa-mir-125b-1, hsa-mir-125b-2, hsa-mir-126, hsa-mir-127, hsa-mir-1307, hsa-mir-139, hsa-mir-140, hsa-mir-141, hsa-mir-142, hsa-mir-143, hsa-mir-145, hsa-mir-148a, hsa-mir-150, hsa-mir-151a, hsa-mir-15b, hsa-mir-17, hsa-mir-181a-1, hsa-mir-181a-2, hsa-mir-182, hsa-mir-183, hsa-mir-191, hsa-mir-192, hsa-mir-193a, hsa-mir 194-1, hsa-mir-196b, hsa-mir-197, hsa-mir-199a-1, hsa-mir-199a-2, hsa-mir-199b, hsa-mir-200c, hsa-mir-203a, hsa-mir-20a, hsa-mir-21, hsa-mir-215, hsa-mir-22, hsa-mir-23a, hsa-mir-23b, hsa-mir-24-1, hsa-mir-24-2, hsa-mir-25, hsa-mir-26a-1, hsa-mir-26a-2, hsa-mir-26b, hsa-mir-27a, hsa-mir-28, hsa-mir-29b-1, hsa-mir-29b-2, hsa-mir-29c, hsa-mir-30d, hsa-mir-30e, hsa-mir-320a, hsa-mir-328, hsa-mir-338, hsa-mir-361, hsa-mir-374a, hsa-mir-375, hsa-mir-378a, hsa-mir-379, hsa-mir-423, hsa-mir-455, hsa-mir-486-1, hsa-mir-486-2, hsa-mir-500a, hsa-mir-574, hsa-mir-625, hsa-mir-92a-1, hsa-mir-92a-2, hsa-mir-92b, hsa-mir-99b | mmu-let-7a-1-3p, mmu-let-7c-1-5p\_+\_1, mmu-let-7c-2-3p, mmu-let-7c-2-5p\_+\_1, mmu-let-7e-5p, mmu-mir-101b-3p, mmu-mir-103-1-3p, mmu-mir-103-2-3p, mmu-mir-106b-3p, mmu-mir-107-3p, mmu-mir-10a-5p, mmu-mir-10a-5p\_+\_1, mmu-mir-125a-5p, mmu-mir-140-3p, mmu-mir-140-3p\_+\_1, mmu-mir-140-5p, mmu-mir-141-3p, mmu-mir-141-5p, mmu-mir-146a-5p, mmu-mir-146b-5p, mmu-mir-148a-3p, mmu-mir-155-5p, mmu-mir-15a-5p, mmu-mir-181d-5p, mmu-mir-182-5p, mmu-mir-183-5p, mmu-mir-183-5p\_+\_1, mmu-mir-185-5p, mmu-mir-192-5p, mmu-mir-192-5p\_+\_1, mmu-mir-194-1-3p, mmu-mir-194-1-5p, mmu-mir-194-2-3p, mmu-mir-194-2-5p, mmu-mir-1981-5p, mmu-mir-19b-1-3p, mmu-mir-19b-2-3p, mmu-mir-200a-3p, mmu-mir-200a-3p\_+\_1, mmu-mir-200c-3p, mmu-mir-203-3p, mmu-mir-203-3p\_+\_1, mmu-mir-215-3p, mmu-mir-215-5p, mmu-mir-215-5p\_+\_1, mmu-mir-21-5p\_-\_1, mmu-mir-218-1-5p, mmu-mir-218-2-5p, mmu-mir-222-3p, mmu-mir-22-3p, mmu-mir-24-1-3p, mmu-mir-24-2-3p, mmu-mir-24-2-5p, mmu-mir-27a-3p, mmu-mir-27b-5p, mmu-mir-29a-3p\_-\_1, mmu-mir-301a-3p, mmu-mir-30b-5p, mmu-mir-30e-5p, mmu-mir-30e-5p\_+\_1, mmu-mir-320-3p, mmu-mir-339-5p, mmu-mir-375-3p, mmu-mir-378-3p, mmu-mir-378-3p\_+\_1, mmu-mir-378-5p, mmu-mir-421-3p, mmu-mir-532-5p, mmu-mir-672-5p, mmu-mir-744-5p, mmu-mir-7b-5p, mmu-mir-802-3p, mmu-mir-802-5p, mmu-mir-872-5p, mmu-mir-92a-2-3p, mmu-mir-99b-3p, mmu-mir-99b-5p |
| TCGA: B (n=3) vs NT (n=8) Mmu Enteroid: B (n=5) vs WT (n=3) | hsa-let-7b, hsa-let-7c, hsa-let-7d, hsa-mir-101-1, hsa-mir-101-2, hsa-mir-10a, hsa-mir-125a, hsa-mir-125b-1, hsa-mir-125b-2, hsa-mir-139, hsa-mir-140, hsa-mir-141, hsa-mir-145, hsa-mir-150, hsa-mir-17, hsa-mir-181a-1, hsa-mir-181a-2, hsa-mir-191, hsa-mir-193a, hsa-mir-197, hsa-mir-203a, hsa-mir-21, hsa-mir-30d, hsa-mir-374a, hsa-mir-423, hsa-mir-486-1, hsa-mir-486-2, hsa-mir-582, hsa-mir-92b | mmu-let-7b-5p, mmu-let-7e-5p, mmu-let-7g-5p, mmu-mir-103-1-3p, mmu-mir-103-2-3p, mmu-mir-106b-3p, mmu-mir-125a-5p, mmu-mir-140-3p\_+\_1, mmu-mir-141-3p, mmu-mir-146b-5p, mmu-mir-155-5p, mmu-mir-181d-5p, mmu-mir-183-5p, mmu-mir-183-5p\_+\_1, mmu-mir-185-5p, mmu-mir-192-5p, mmu-mir-192-5p\_+\_1, mmu-mir-194-1-3p, mmu-mir-194-1-5p, mmu-mir-194-2-5p, mmu-mir-1981-5p, mmu-mir-200a-3p, mmu-mir-200c-3p, mmu-mir-203-3p, mmu-mir-203-3p\_+\_1, mmu-mir-21-3p, mmu-mir-215-3p, mmu-mir-215-5p, mmu-mir-215-5p\_+\_1, mmu-mir-21-5p\_-\_1, mmu-mir-218-1-5p, mmu-mir-218-2-5p, mmu-mir-222-3p, mmu-mir-22-3p, mmu-mir-24-2-5p, mmu-mir-25-3p, mmu-mir-26a-1-5p, mmu-mir-26a-2-5p, mmu-mir-30a-3p, mmu-mir-30e-5p, mmu-mir-30e-5p\_+\_1, mmu-mir-320-3p, mmu-mir-340-5p, mmu-mir-345-3p\_-\_1, mmu-mir-374-5p, mmu-mir-378-3p, mmu-mir-378-3p\_+\_1, mmu-mir-378-5p, mmu-mir-421-3p, mmu-mir-425-5p, mmu-mir-672-5p, mmu-mir-7b-5p, mmu-mir-802-3p, mmu-mir-802-5p, mmu-mir-872-5p, mmu-mir-99b-3p, mmu-mir-99b-5p |
| TCGA: KP (n=9) vs NT (n=8) Mmu Enteroid: KRP (n=2) vs WT (n=3) | hsa-let-7a-1, hsa-let-7a-2, hsa-let-7a-3, hsa-let-7b, hsa-let-7c, hsa-let-7d, hsa-let-7f-1, hsa-let-7f-2, hsa-let-7i, hsa-mir-101-1, hsa-mir-101-2, hsa-mir-10a, hsa-mir-10b, hsa-mir-125a, hsa-mir-125b-1, hsa-mir-125b-2, hsa-mir-126, hsa-mir-127, hsa-mir-1307, hsa-mir-139, hsa-mir-140, hsa-mir-141, hsa-mir-142, hsa-mir-143, hsa-mir-145, hsa-mir-146b, hsa-mir-148a, hsa-mir-150, hsa-mir-151a, hsa-mir-15b, hsa-mir-17, hsa-mir-181a-1, hsa-mir-181a-2, hsa-mir-182, hsa-mir-183, hsa-mir-191, hsa-mir-192, hsa-mir-193a, hsa-mir-197, hsa-mir-199a-1, hsa-mir-199a-2, hsa-mir-199b, hsa-mir-200c, hsa-mir-203a, hsa-mir-21, hsa-mir-215, hsa-mir-22, hsa-mir-24-1, hsa-mir-24-2, hsa-mir-26a-1, hsa-mir-26a-2, hsa-mir-26b, hsa-mir-27a, hsa-mir-28, hsa-mir-29c, hsa-mir-30d, hsa-mir-30e, hsa-mir-320a, hsa-mir-328, hsa-mir-361, hsa-mir-374a, hsa-mir-375, hsa-mir-378a, hsa-mir-379, hsa-mir-423, hsa-mir-486-1, hsa-mir-486-2, hsa-mir-500a, hsa-mir-574, hsa-mir-582, hsa-mir-625, hsa-mir-92a-1, hsa-mir-92a-2, hsa-mir-92b, hsa-mir-99b | mmu-let-7b-5p, mmu-let-7d-3p, mmu-mir-103-1-3p, mmu-mir-103-2-3p, mmu-mir-130a-3p, mmu-mir-141-3p, mmu-mir-146a-5p, mmu-mir-148a-3p, mmu-mir-155-5p, mmu-mir-181a-1-5p, mmu-mir-181a-2-5p, mmu-mir-194-1-5p, mmu-mir-1981-5p, mmu-mir-200c-3p, mmu-mir-205-5p, mmu-mir-21-3p, mmu-mir-21-5p, mmu-mir-21-5p\_-\_1, mmu-mir-21-5p\_+\_1, mmu-mir-21-5p\_+\_2, mmu-mir-24-2-5p, mmu-mir-26a-1-5p, mmu-mir-26a-2-5p, mmu-mir-26b-5p, mmu-mir-27a-3p, mmu-mir-28-3p, mmu-mir-3096b-5p, mmu-mir-3096b-5p\_-\_1, mmu-mir-30a-3p, mmu-mir-30a-3p\_+\_1, mmu-mir-30a-5p, mmu-mir-30b-5p, mmu-mir-30d-5p, mmu-mir-30e-3p, mmu-mir-30e-5p, mmu-mir-320-3p, mmu-mir-339-5p, mmu-mir-340-5p, mmu-mir-34a-5p, mmu-mir-378-3p, mmu-mir-378-3p\_+\_1, mmu-mir-378-5p, mmu-mir-674-5p, mmu-mir-7a-1-5p, mmu-mir-7a-2-5p, mmu-mir-7b-5p, mmu-mir-99b-3p, mmu-mir-99b-5p |
| TCGA: KS (n=2) vs NT (n=8) Mmu Enteroid: KRS (n=3) vs WT (n=3) | hsa-mir-181a-1, hsa-let-7a-2, hsa-let-7a-1, hsa-let-7a-3, hsa-let-7f-1, hsa-let-7f-2, hsa-mir-24-1, hsa-mir-24-2, hsa-mir-223, hsa-mir-143, hsa-mir-30b, hsa-mir-379 | mmu-let-7a-1-3p, mmu-let-7c-2-3p, mmu-let-7e-5p, mmu-mir-103-1-3p, mmu-mir-103-2-3p, mmu-mir-106b-3p, mmu-mir-107-3p, mmu-mir-125a-5p, mmu-mir-128-1-3p, mmu-mir-128-2-3p, mmu-mir-140-3p\_+\_1, mmu-mir-146b-5p, mmu-mir-148a-3p, mmu-mir-155-5p, mmu-mir-15b-5p, mmu-mir-16-1-5p, mmu-mir-16-2-5p, mmu-mir-17-5p, mmu-mir-181a-1-5p, mmu-mir-181a-2-5p, mmu-mir-181b-1-5p, mmu-mir-181b-2-5p, mmu-mir-182-5p, mmu-mir-183-5p, mmu-mir-183-5p\_+\_1, mmu-mir-190-5p, mmu-mir-192-5p, mmu-mir-192-5p\_+\_1, mmu-mir-194-1-3p, mmu-mir-194-1-5p, mmu-mir-194-2-3p, mmu-mir-194-2-5p, mmu-mir-1981-5p, mmu-mir-19a-3p, mmu-mir-203-3p, mmu-mir-203-3p\_+\_1, mmu-mir-205-5p, mmu-mir-20a-5p, mmu-mir-21-3p, mmu-mir-215-3p, mmu-mir-215-5p, mmu-mir-215-5p\_+\_1, mmu-mir-21-5p, mmu-mir-21-5p\_-\_1, mmu-mir-21-5p\_+\_1, mmu-mir-21-5p\_+\_2, mmu-mir-218-1-5p, mmu-mir-218-2-5p, mmu-mir-221-3p, mmu-mir-222-3p, mmu-mir-24-2-5p, mmu-mir-27a-3p, mmu-mir-29a-3p\_-\_1, mmu-mir-30a-3p, mmu-mir-30a-3p\_+\_1, mmu-mir-30a-5p, mmu-mir-30a-5p\_+\_1, mmu-mir-375-3p, mmu-mir-375-3p\_-\_1, mmu-mir-378-3p, mmu-mir-378-3p\_+\_1, mmu-mir-378-5p, mmu-mir-421-3p, mmu-mir-672-5p, mmu-mir-7b-5p, mmu-mir-802-3p, mmu-mir-802-5p, mmu-mir-872-5p, mmu-mir-92a-2-3p, mmu-mir-99b-3p, mmu-mir-99b-5p |
| TCGA: KPS (n=2) vs NT (n=8) Mmu Enteroid: KRPS (n=4) vs WT (n=3) | hsa-let-7c, hsa-let-7d, hsa-let-7f-1, hsa-let-7f-2, hsa-mir-106b, hsa-mir-10a, hsa-mir-143, hsa-mir-151a, hsa-mir-15b, hsa-mir-197, hsa-mir-200c, hsa-mir-30e, hsa-mir-328, hsa-mir-374a, hsa-mir-486-1, hsa-mir-486-2, hsa-mir-99b | mmu-let-7b-5p, mmu-let-7c-1-5p, mmu-let-7c-2-5p, mmu-let-7d-3p, mmu-let-7e-5p, mmu-let-7g-5p, mmu-let-7i-5p, mmu-mir-101b-3p\_-\_1, mmu-mir-103-1-3p, mmu-mir-103-2-3p, mmu-mir-106b-5p, mmu-mir-10a-5p, mmu-mir-125a-5p, mmu-mir-140-3p, mmu-mir-140-3p\_+\_1, mmu-mir-141-3p, mmu-mir-141-5p, mmu-mir-146a-5p, mmu-mir-146b-5p, mmu-mir-148a-3p, mmu-mir-151-3p, mmu-mir-155-5p, mmu-mir-15a-5p, mmu-mir-183-5p, mmu-mir-183-5p\_+\_1, mmu-mir-1839-5p, mmu-mir-185-5p, mmu-mir-186-5p, mmu-mir-18a-5p, mmu-mir-190-5p, mmu-mir-191-5p, mmu-mir-192-5p, mmu-mir-192-5p\_+\_1, mmu-mir-194-1-3p, mmu-mir-194-1-5p, mmu-mir-194-2-3p, mmu-mir-194-2-5p, mmu-mir-1981-5p, mmu-mir-200a-3p\_+\_2, mmu-mir-200b-5p, mmu-mir-200c-3p, mmu-mir-203-3p, mmu-mir-203-3p\_+\_1, mmu-mir-205-5p, mmu-mir-21-3p, mmu-mir-215-3p, mmu-mir-215-5p, mmu-mir-215-5p\_+\_1, mmu-mir-21-5p, mmu-mir-21-5p\_-\_1, mmu-mir-21-5p\_+\_1, mmu-mir-21-5p\_+\_2, mmu-mir-21-5p\_+\_4, mmu-mir-218-1-5p, mmu-mir-218-2-5p, mmu-mir-221-3p, mmu-mir-222-3p, mmu-mir-23a-3p, mmu-mir-24-1-3p, mmu-mir-24-2-3p, mmu-mir-24-2-5p, mmu-mir-25-3p, mmu-mir-26a-1-5p, mmu-mir-26a-2-5p, mmu-mir-26b-5p\_-\_1, mmu-mir-27a-3p, mmu-mir-27b-5p, mmu-mir-28-3p, mmu-mir-29a-3p\_-\_1, mmu-mir-301a-3p, mmu-mir-301b-3p, mmu-mir-3096-5p, mmu-mir-3096-5p\_+\_1, mmu-mir-3096b-5p, mmu-mir-3096b-5p\_-\_1, mmu-mir-30a-5p\_+\_1, mmu-mir-30b-5p, mmu-mir-30d-5p, mmu-mir-30e-3p, mmu-mir-31-5p, mmu-mir-320-3p, mmu-mir-328-3p, mmu-mir-339-5p, mmu-mir-340-5p, mmu-mir-34a-5p, mmu-mir-374-5p,mmu-mir-375-3p, mmu-mir-375-3p\_-\_1, mmu-mir-378-3p, mmu-mir-378-3p\_+\_1, mmu-mir-378-5p, mmu-mir-421-3p, mmu-mir-423-3p, mmu-mir-423-3p\_-\_1, mmu-mir-423-5p, mmu-mir-425-5p, mmu-mir-672-5p,mmu-mir-674-5p, mmu-mir-744-5p,mmu-mir-7a-1-5p, mmu-mir-7a-2-5p, mmu-mir-7b-5p, mmu-mir-802-3p, mmu-mir-802-5p, mmu-mir-872-5p, mmu-mir-92a-1-3p, mmu-mir-92a-1-3p\_+\_1, mmu-mir-92a-2-3p, mmu-mir-99b-3p, mmu-mir-99b-5p |

## Slide 4
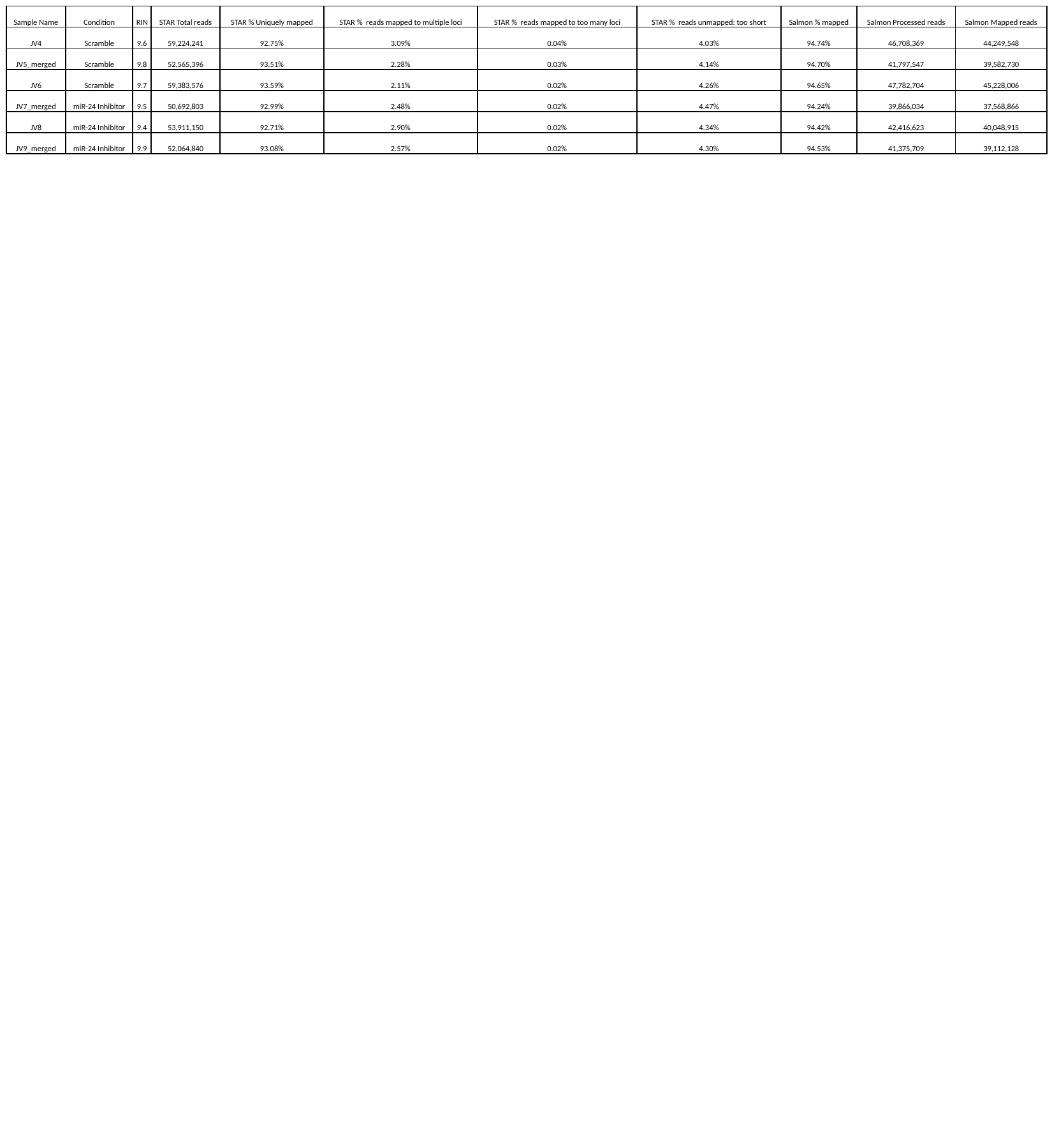

| Sample Name | Condition | RIN | STAR Total reads | STAR % Uniquely mapped | STAR % reads mapped to multiple loci | STAR % reads mapped to too many loci | STAR % reads unmapped: too short | Salmon % mapped | Salmon Processed reads | Salmon Mapped reads |
| --- | --- | --- | --- | --- | --- | --- | --- | --- | --- | --- |
| JV4 | Scramble | 9.6 | 59,224,241 | 92.75% | 3.09% | 0.04% | 4.03% | 94.74% | 46,708,369 | 44,249,548 |
| JV5\_merged | Scramble | 9.8 | 52,565,396 | 93.51% | 2.28% | 0.03% | 4.14% | 94.70% | 41,797,547 | 39,582,730 |
| JV6 | Scramble | 9.7 | 59,383,576 | 93.59% | 2.11% | 0.02% | 4.26% | 94.65% | 47,782,704 | 45,228,006 |
| JV7\_merged | miR-24 Inhibitor | 9.5 | 50,692,803 | 92.99% | 2.48% | 0.02% | 4.47% | 94.24% | 39,866,034 | 37,568,866 |
| JV8 | miR-24 Inhibitor | 9.4 | 53,911,150 | 92.71% | 2.90% | 0.02% | 4.34% | 94.42% | 42,416,623 | 40,048,915 |
| JV9\_merged | miR-24 Inhibitor | 9.9 | 52,064,840 | 93.08% | 2.57% | 0.02% | 4.30% | 94.53% | 41,375,709 | 39,112,128 |

## Slide 5
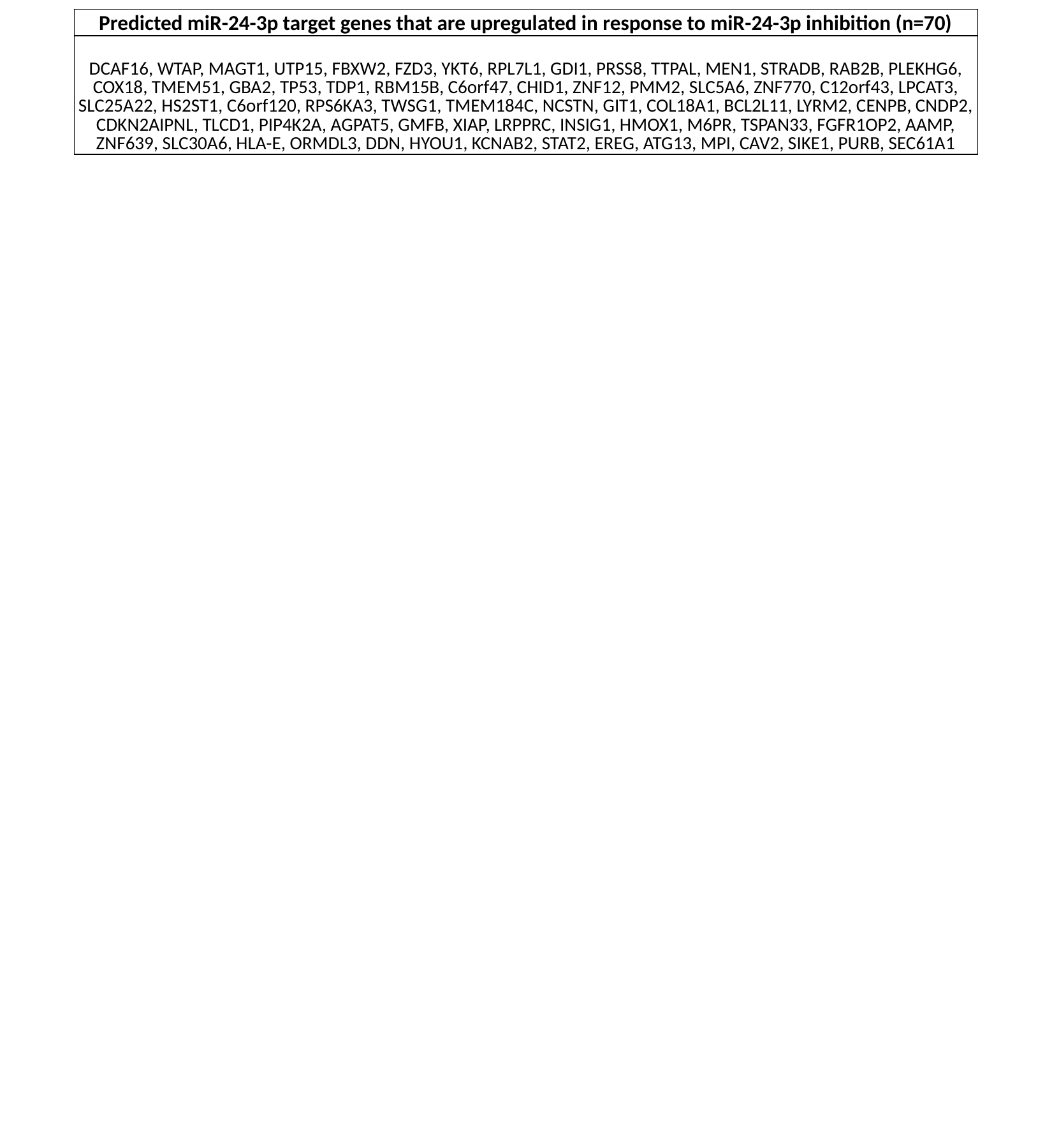

| Predicted miR-24-3p target genes that are upregulated in response to miR-24-3p inhibition (n=70) |
| --- |
| DCAF16, WTAP, MAGT1, UTP15, FBXW2, FZD3, YKT6, RPL7L1, GDI1, PRSS8, TTPAL, MEN1, STRADB, RAB2B, PLEKHG6, COX18, TMEM51, GBA2, TP53, TDP1, RBM15B, C6orf47, CHID1, ZNF12, PMM2, SLC5A6, ZNF770, C12orf43, LPCAT3, SLC25A22, HS2ST1, C6orf120, RPS6KA3, TWSG1, TMEM184C, NCSTN, GIT1, COL18A1, BCL2L11, LYRM2, CENPB, CNDP2, CDKN2AIPNL, TLCD1, PIP4K2A, AGPAT5, GMFB, XIAP, LRPPRC, INSIG1, HMOX1, M6PR, TSPAN33, FGFR1OP2, AAMP, ZNF639, SLC30A6, HLA-E, ORMDL3, DDN, HYOU1, KCNAB2, STAT2, EREG, ATG13, MPI, CAV2, SIKE1, PURB, SEC61A1 |

## Slide 6
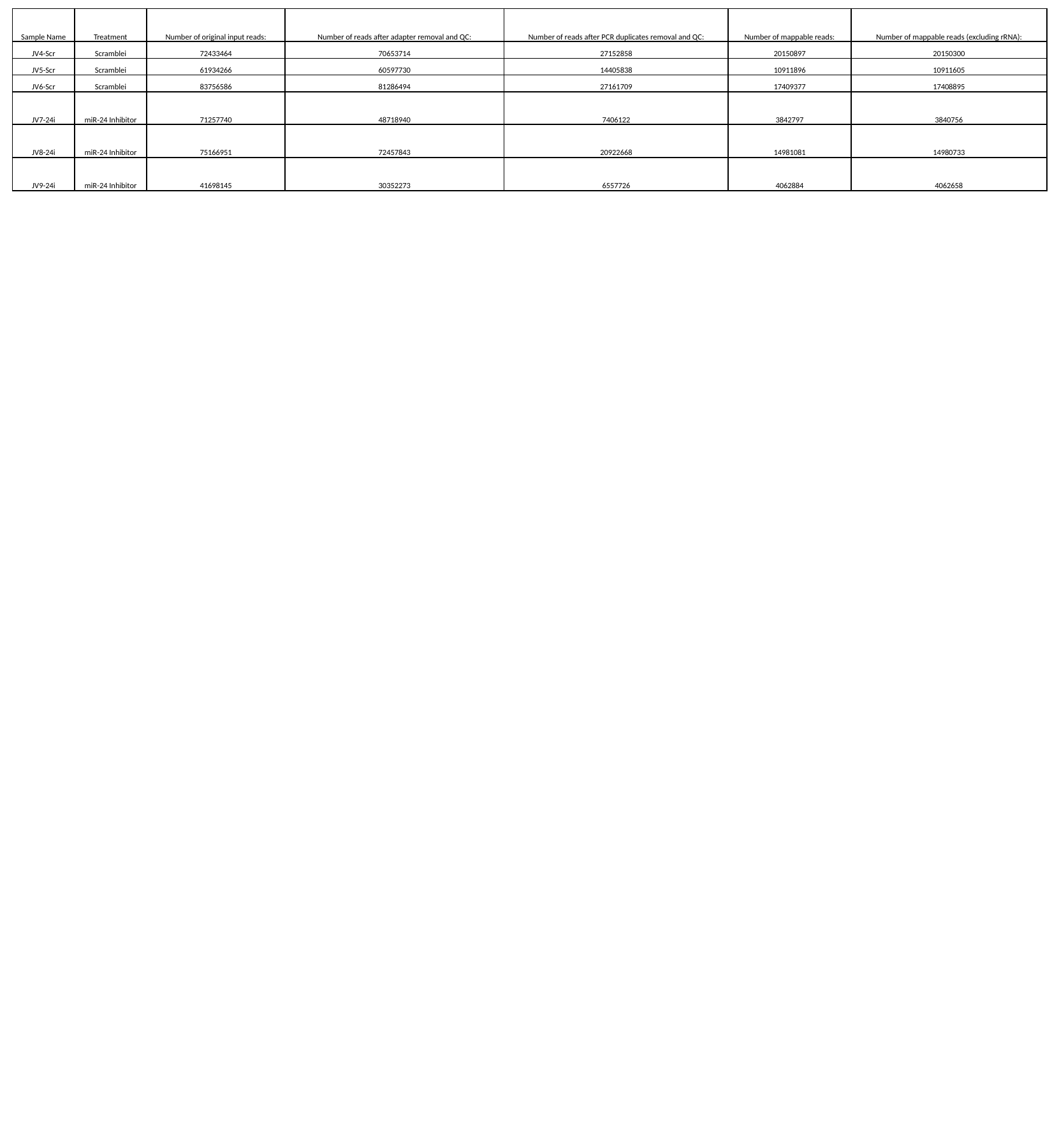

| Sample Name | Treatment | Number of original input reads: | Number of reads after adapter removal and QC: | Number of reads after PCR duplicates removal and QC: | Number of mappable reads: | Number of mappable reads (excluding rRNA): |
| --- | --- | --- | --- | --- | --- | --- |
| JV4-Scr | Scramblei | 72433464 | 70653714 | 27152858 | 20150897 | 20150300 |
| JV5-Scr | Scramblei | 61934266 | 60597730 | 14405838 | 10911896 | 10911605 |
| JV6-Scr | Scramblei | 83756586 | 81286494 | 27161709 | 17409377 | 17408895 |
| JV7-24i | miR-24 Inhibitor | 71257740 | 48718940 | 7406122 | 3842797 | 3840756 |
| JV8-24i | miR-24 Inhibitor | 75166951 | 72457843 | 20922668 | 14981081 | 14980733 |
| JV9-24i | miR-24 Inhibitor | 41698145 | 30352273 | 6557726 | 4062884 | 4062658 |

## Slide 7
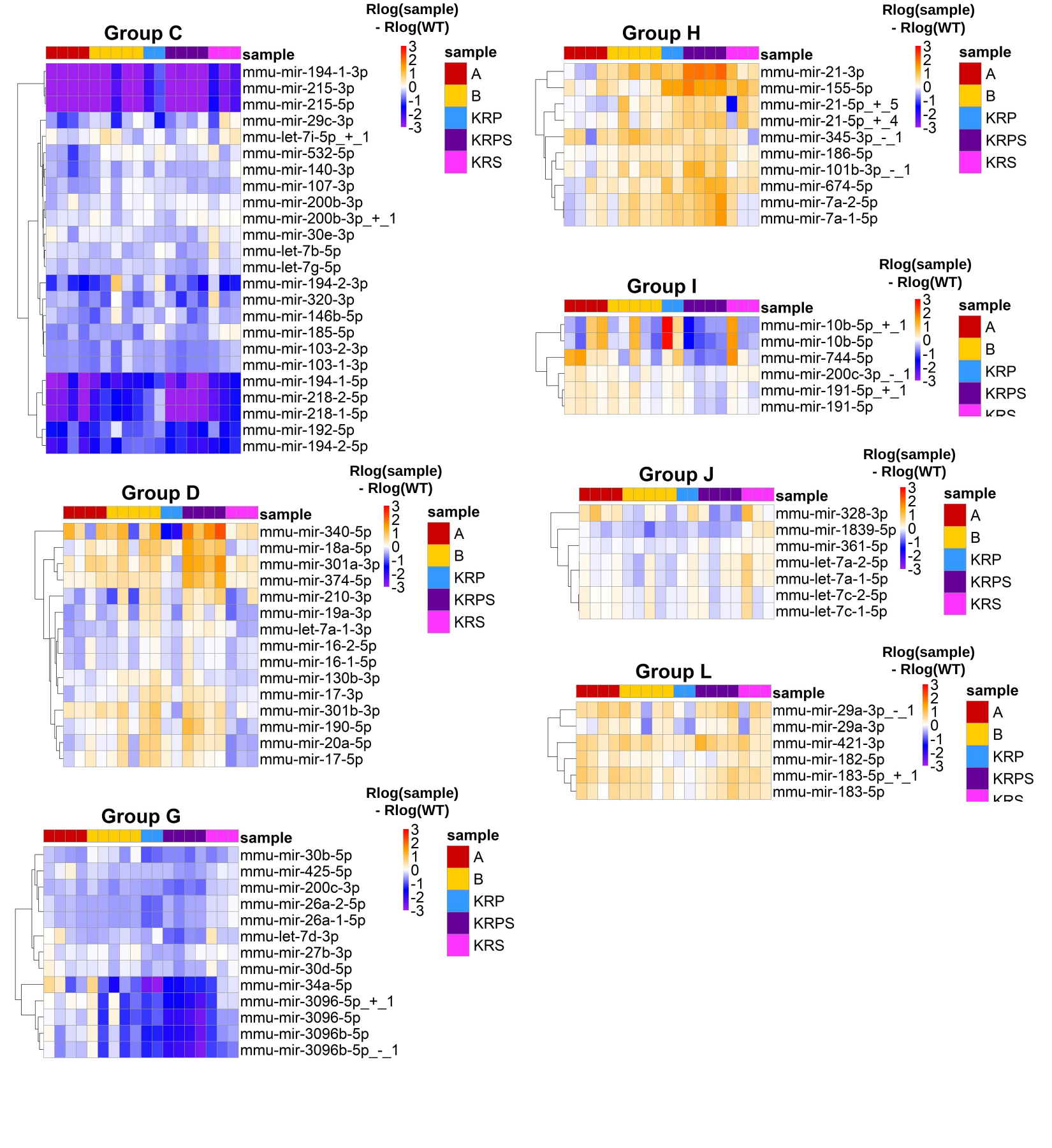

Rlog(sample) - Rlog(WT)
Rlog(sample) - Rlog(WT)
Rlog(sample) - Rlog(WT)
Rlog(sample) - Rlog(WT)
Rlog(sample) - Rlog(WT)
Rlog(sample) - Rlog(WT)
Rlog(sample) - Rlog(WT)

## Slide 8
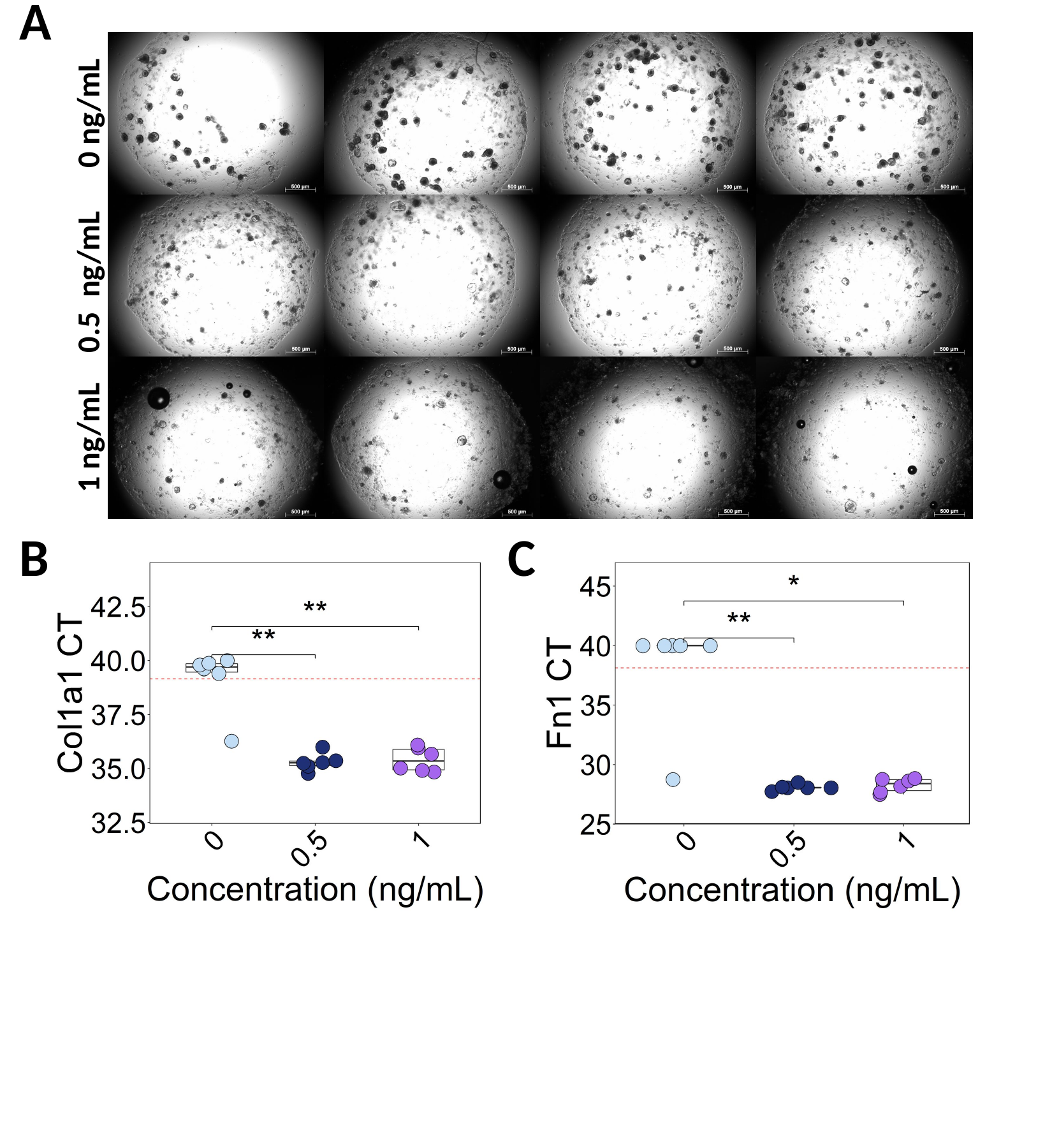

A
0 ng/mL
0.5 ng/mL
1 ng/mL
C
B

## Slide 9
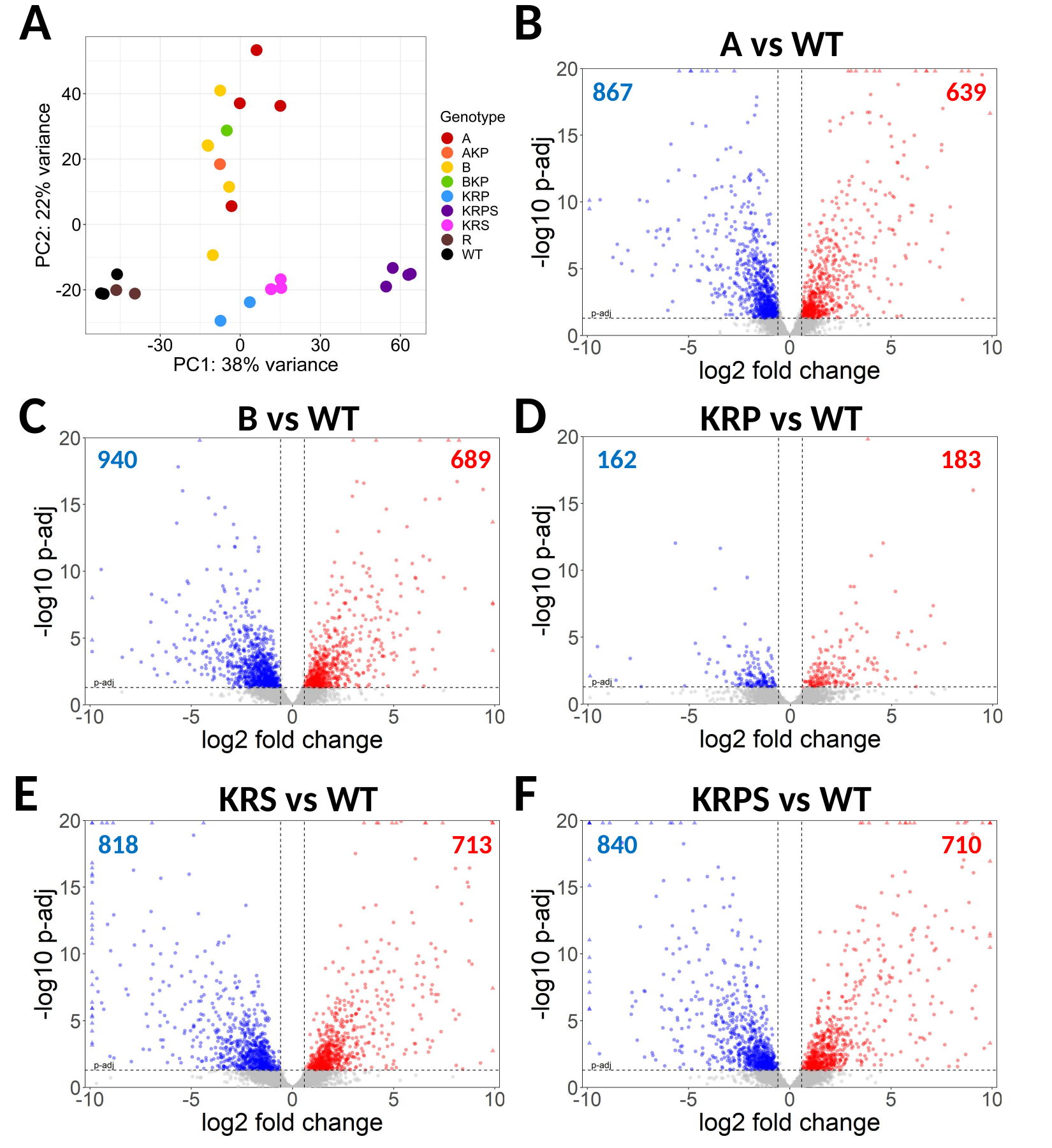

A
B
A vs WT
867
639
C
D
B vs WT
KRP vs WT
162
183
940
689
E
F
KRS vs WT
KRPS vs WT
840
710
818
713

## Slide 10
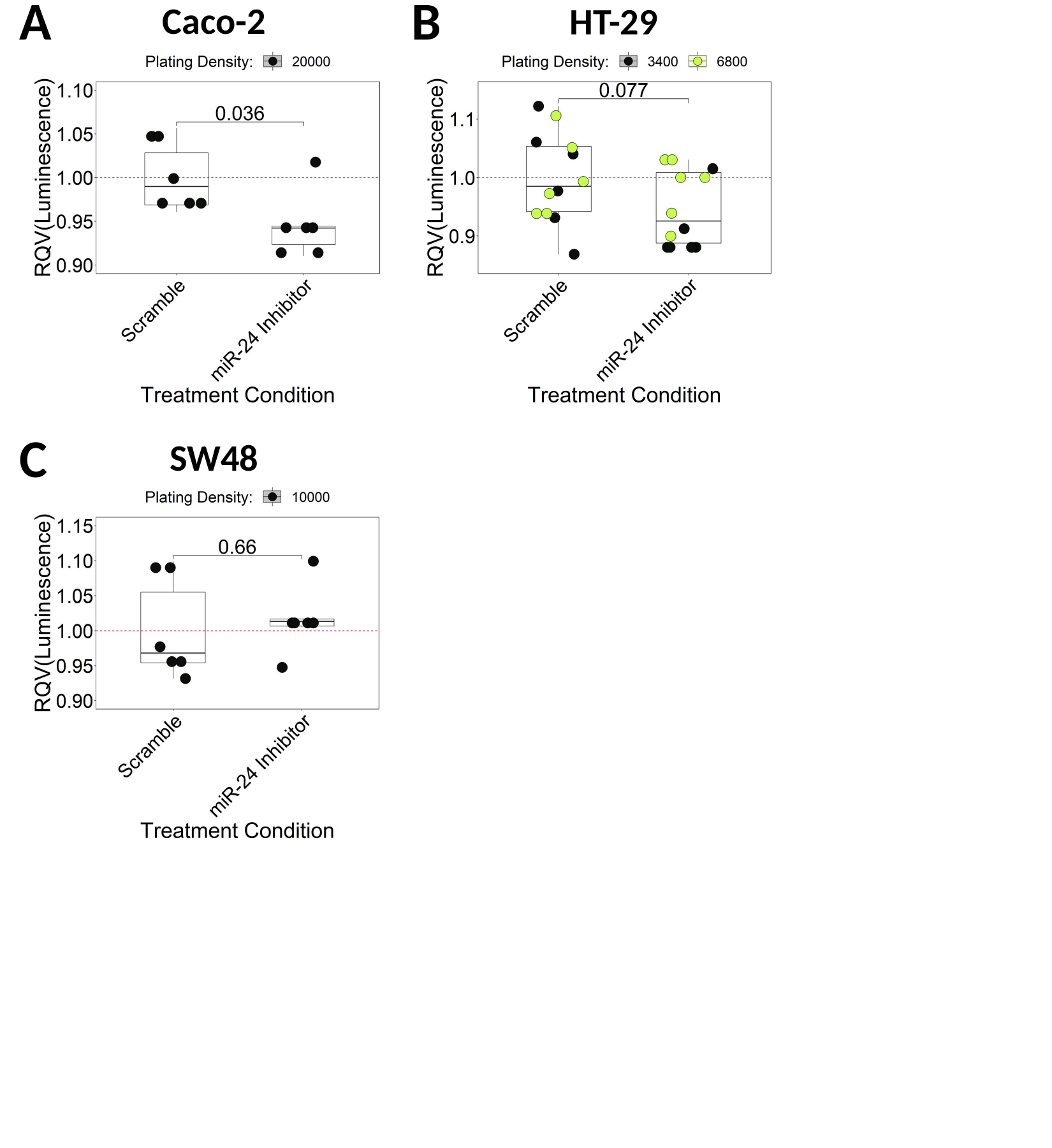

Supplement Figure 4
A
B
Caco-2
HT-29
C
SW48

## Slide 11
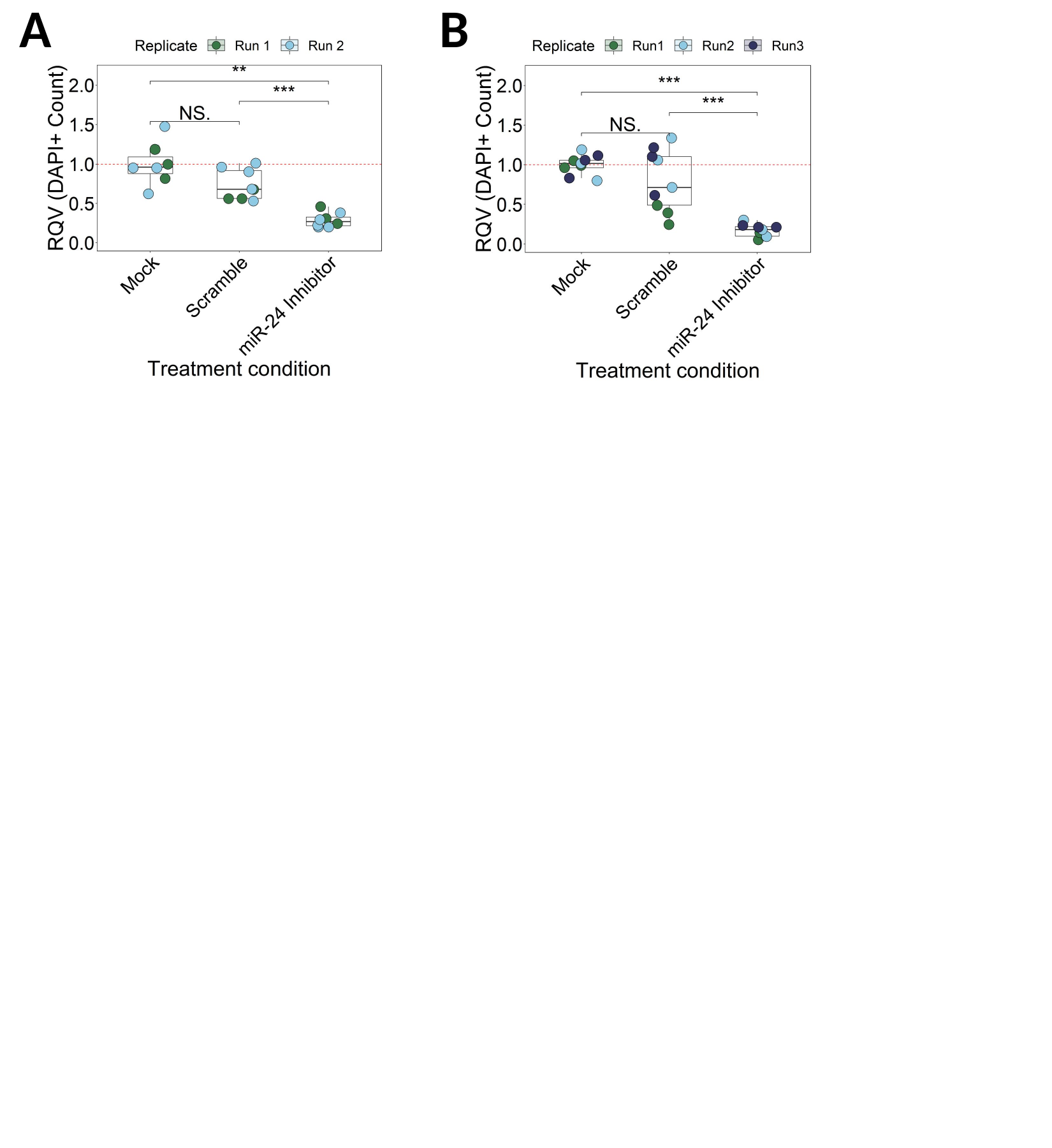

A
B

## Slide 12
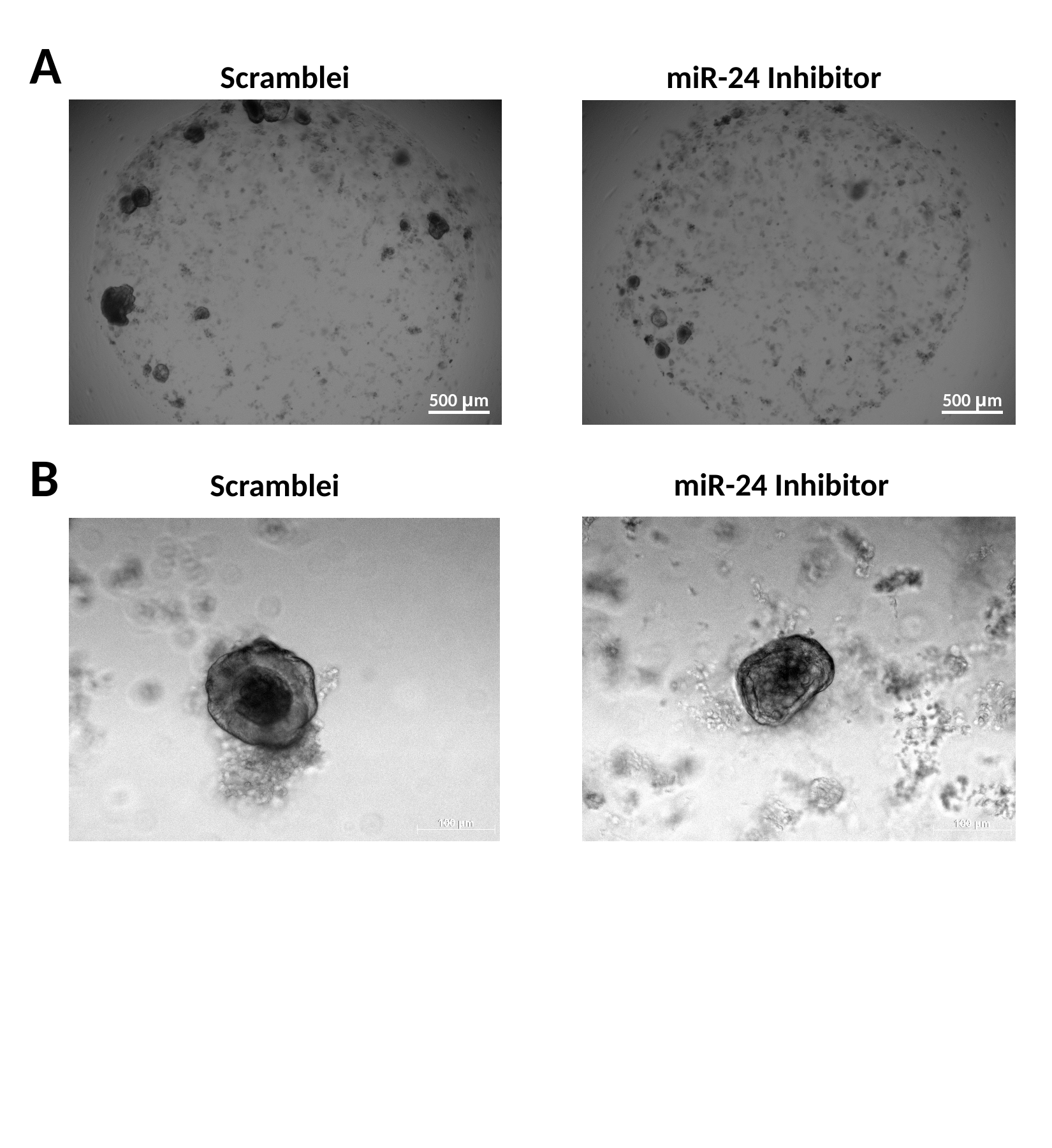

A
miR-24 Inhibitor
Scramblei
500 μm
500 μm
B
miR-24 Inhibitor
Scramblei

## Slide 13
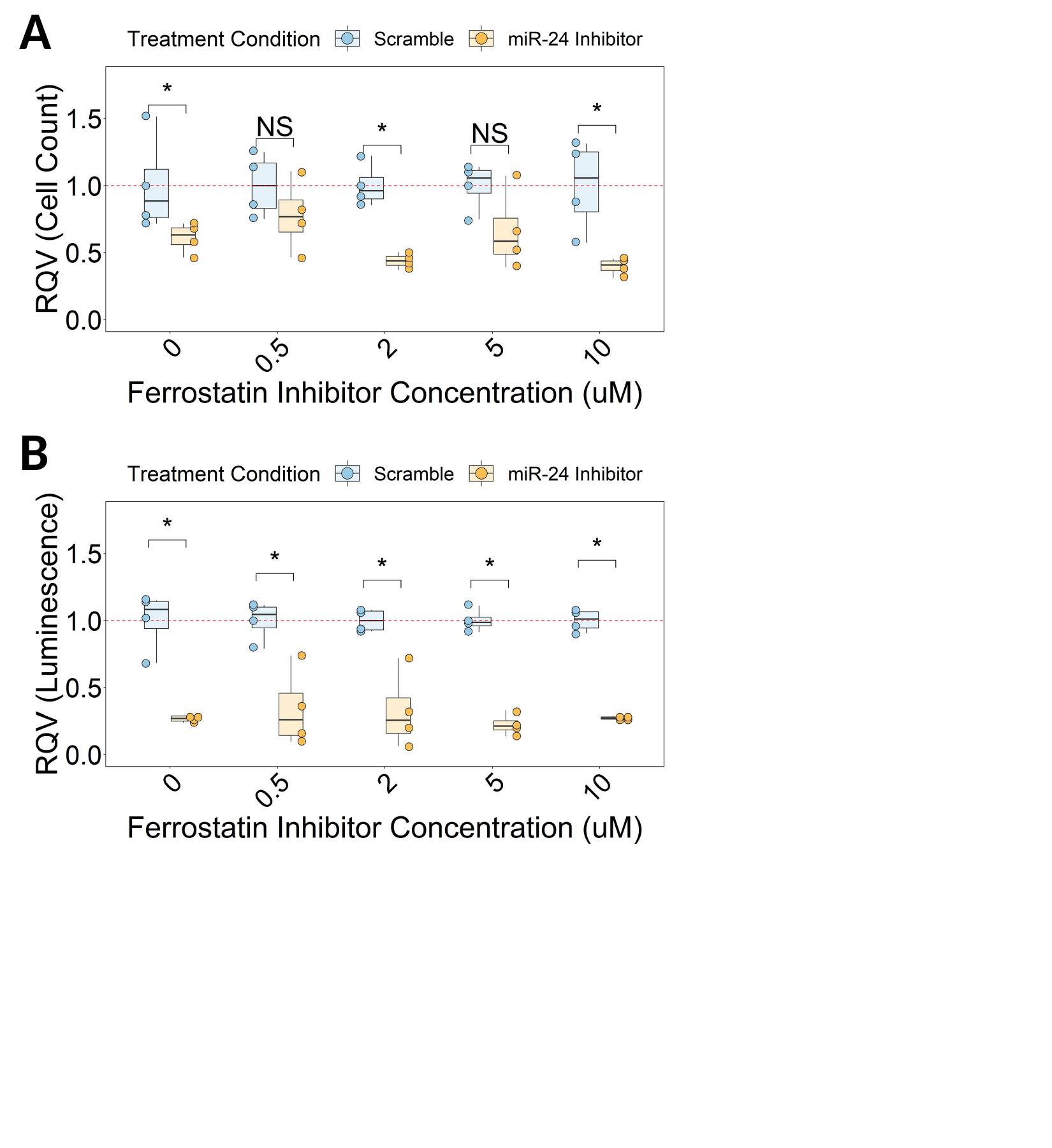

A
B
